# Respect Our Identity

**Published:** 1883-12

**Authors:** 


### RESPECT OUR IDENTITY.

The editor of the *Missouri Dental Journal*, in accrediting articles, confounds the INDEPENDENT PRACTITIONER with the *Dental Practitioner*. It was the latter that published Dr. L. Ashley Faught's article on "The Business Qualifications of Professional Men." The INDEPENDENT PRACTITIONER does not desire to be held responsible for the presentation of such sentiments.

